# Historical dynamics of the Chinese dynasties

**DOI:** 10.1016/j.heliyon.2021.e07293

**Published:** 2021-06-15

**Authors:** Sabin Roman

**Affiliations:** Centre for the Study of Existential Risk, University of Cambridge, United Kingdom

**Keywords:** Chaotic map, Chinese dynasties, Territorial division, Mathematical modelling, History

## Abstract

We develop a framework for studying state division and unification, and as a case study we focus on modelling the territorial patterns in imperial China during periods of unity and upheaval. As a modelling tool we employ discrete dynamical systems and analyse two models: the logistic map and a new class of maps, which we name ren maps. The critical transitions exhibited by the models can be used to capture the process of territorial division but also unification. We outline certain limitations of uni-modal, smooth maps for our modelling purposes and propose ren maps as an alternative, which we use to reproduce the territorial dynamics over time. As a result of the modelling we arrive at a quantitative measure for asabiyyah, a notion of group solidarity, whose secular cycles match the historical record over 1800 years, from the time of the Warring States to the beginning of the Ming dynasty. Furthermore, we also derive an equation for aggregate asabiyyah which can be employed in other cases of interest.

## 1 Introduction

Lorenz discovered chaos in its modern mathematical sense when he analyzed a set of ordinary differential equations derived from a model of thermal convection (Lorenz, 1963). Ever since, chaos theory has been a focal point of research in dynamical systems, with sustained efforts to apply its insights in the case of social phenomena (Kiel and Elliott, 1996; Robertson and Combs, 2014), but finding robust patterns has proven challenging. Nevertheless, significant progress has been made in modelling social dynamics using ordinary differential equations, in particular for ancient societies (Brander and Taylor, 1998; Roman et al., 2017; Kuil et al., 2016; Roman et al., 2018; Anderies, 1998; Roman and Palmer, 2019), but discrete systems or maps have rarely been employed despite showing rich dynamical behaviour, including chaotic behaviour in one dimension (May, 1976; Feigenbaum, 1978). We present a mathematical framework for modelling alternating periods of stability and disorder using discrete dynamical systems. Our focus is on reproducing the territorial changes seen throughout the imperial period 221 BCE-1912 CE of Chinese history. The history of China has known a number of periods of increased stability, such as during the Han (206 BCE-220 CE), Tang (618 CE-907 CE) and later dynasties (Gernet, 1996; Dardess, 2010) as well as turbulent times, which was the case during the period of the Sixteen Kingdoms (304 CE-439 CE) or of the Five Dynasties and Ten Kingdoms (907 CE-979 CE). The change of dynasties and the alternating cycle between periods of stability and upheaval has led to several theories attempting to explain these historical patterns, a well-known example being the dynastic cycle (Meskill, 1965; Elvin, 1973).

We investigate if the transition between the different periods can be modelled as bifurcations of discrete maps. We analyse and compare two models, assessing their strengths and weaknesses in capturing the features present in the historical data that tracks the areas of the territories controlled by the different dynasties (Taagepera, 1979, Taagepera, 1997; Song, 1994; Tan, 1982). Furthermore, starting from the models, we aim to quantify the degree of asabiyyah (Khaldun, 1958), a notion of group solidarity that been used to explain state breakdown (Turchin, 2018), and determine its long-term trend over time. We use the simplified writing “asabiya”, which is common in the mathematical treatments of the topic (Turchin, 2018).

In section 2 we review the mathematical models of the dynastic cycle, the majority of which focus on demographic aspects of the regime change. In section 3 we introduce the two models that we consider: the logistic map (May, 1976), which is representative of all uni-modal, smooth maps, and a new class of maps, which we refer to as ren maps, named after the Chinese symbol. We explicitly model the historical record using ren maps, which we show can provide a coarse description of the evolution of the system. The transition between order and disorder in the historical record is modelled as a transition between a stable fixed point, corresponding to the dominant dynasty of a peaceful era, and a chaotic regime (in a mathematical sense), where multiple competing factions are present.

The structure of the models suggests a way to quantify the degree of social solidarity, or asabiya, seen throughout history of imperial China and in section 4 we propose an equation for asabiya that contrasts with prior research. We also analyze and compare our estimate of asabiya with population data (Zhao and Xie, 1988). Related trends emerge which indicate the presence of a feedback mechanism between territorial integrity, population dynamics and the extent of social solidarity.

## 2 Literature review

Given the vast literature pertaining to the Chinese dynastic cycle, we only review work with similar methods and scope as our own, namely: that develops a mathematical model, aims to conceptually or numerically match the model to Chinese history or provide a mathematical definition for otherwise qualitative concepts (e.g., asabiya).

Certain mathematical models have been developed to quantify hypothesized mechanisms underlying the dynastic cycles. A common framework for the models is to divide the population into farmers, bandits and rulers. If the population is too large compared to available food, then peasants can turn to banditry and with the reduction in farmers and increased theft, revenue from taxation is strained, making it difficult to finance the army and civil service (Yu-Ch'uan, 1936; Meskill, 1965; Skinner, 1985). Thus, the dynasty can fall, and population decrease substantially. With a reduced population, then a new dynasty can appear, and the process is started again.

Early work (Usher, 1989) build a model that aims to capture the above pattern but also adds the neoclassic economic assumption of utility maximization. The model is specified by 14 equations, but no numerical values are specified for the parameters, nor is any solution to the equations compared with real data. Later work (Chu and Lee, 1994) formalizes a similar narrative into several utility-maximizing, econometric models with different assumptions on how population changes. The predictions of the models are compared with historical data on population growth (Zhao and Xie, 1988), and one model performs notably well. The general dynamic of bandits and farmers within the model is akin to predator-prey interactions, and the findings overall support the hypothetical causal relations of the dynastic cycle as described above. Recent work (Chan and Laffargue, 2016) has also employed economic modelling through utility maximization.

Other work (Feichtinger and Novak, 1994) modelled historical relationships as a differential game, where a periodic Nash equilibrium is possible, with wealth oscillating between the state and the bandits. A different model (Feichtinger et al., 1996), that does not account for any explicit decision making, proposes a system of ordinary differential equations (ODEs) that model the dynamics of farmers, bandits and rulers. The model structure is analogous to food-chains in ecology, with the farmers acting as prey, bandits as predators and rulers as super-predators, that exploit both “species”. More recent work (Saeed and Pavlov, 2008) has also developed an ODE model; in contrast to prior research, the model accounts for the constrained capacity that is being appropriated between alternative uses.

The work in (Usher, 1989; Chu and Lee, 1994; Feichtinger and Novak, 1994; Chan and Laffargue, 2016) relies on the assumption of utility maximization by the individuals in the population, which has faced criticism on general grounds (Rubinstein, 1998; Urbina and Ruiz-Villaverde, 2019), and in the particular case of modelling the dynastic cycle where “a sociologically more sophisticated approach is needed that would build upon collectively held norms and collectively made decisions” (Turchin, 2018, p. 138). Later contributions (Feichtinger et al., 1996; Saeed and Pavlov, 2008) focus on developing dynamical models using a more heuristic approach, but limit themselves to a theoretical analysis (e.g., analyzing attractor states) and do not attempt a comparison with historical data for empirical validation. In addition, the emphasis on popular rebellion as a mechanism causing state collapse is problematic because peasants are not adequately armed, trained or organized to bring down the state. Rather, factional fighting between elites can led to state collapse and facilitate popular rebellions, which are often led by members of the elite (Goldstone, 2016).

A growing volume of recent research has focused on analyzing the impact of climate change on the dynastic cycle and on the demographic trends in Chinese history (Zhang et al., 2005, Zhang et al., 2006; Fan, 2010; Lee and Zhang, 2010; Fan, 2015; Chen, 2015; Wei et al., 2015; Yin et al., 2016). This work complements the Malthusian perspective on population pressure and provides evidence of important exogenous drivers on societal development. Our results do not exclude such exogenous contributions to societal dynamics but our main emphasis has been on determining the relationship between dynastic configurations and difference in territory (Yang, 1954).

In the present work we depart from farmer-bandit-state framework and focus on modelling territorial dynamics and comparing it with global demographic trends, but not pertaining to specializations of the population. Furthermore, we aim to quantify the degree of asabiya and how it evolves over time in the case of the Chinese dynasties. Similar work has been done (Turchin, 2018) which builds an ODE model of territorial changes and asabiya growth that aims to quantify qualitative insights on the interplay between these two variables. The model is illustrative in nature and is not applied to any case study. But an extension of the model that incorporates spatial dynamics is also developed and uses a grid of 21x21 cells, with two equations for each cell. Asabiya in each cell follows the Verhulst logistic equation (Bacaër, 2011) in the frontier regions and decays exponentially otherwise. The model produces patterns similar to historical trajectories of polities in Central and East Asia between the 6th and 13th centuries.

We propose an alternative framework for modelling the territorial evolution. The mathematical structure we employ in our models are one-dimensional discrete dynamical systems, also known as iterated functions or maps. These can show rich dynamical behaviour, including chaotic behaviour in one dimension. By considering different maps and comparing their attractor states, our goal is to fit the historical record (Taagepera, 1979, Taagepera, 1997; Song, 1994; Tan, 1982), aiming to reproduce periods of fragmentation and unification. We then determine the levels of asabiya from the fit of the model to the historical record and deduce a general, mean-field equation governing the macro-evolution of asabiya for the Chinese dynasties. Thus, the equation for asabiya is inferred from the archaeological record and not a starting point of the model, but rather an outcome.

## 3 Discrete maps

**Figure 1:**
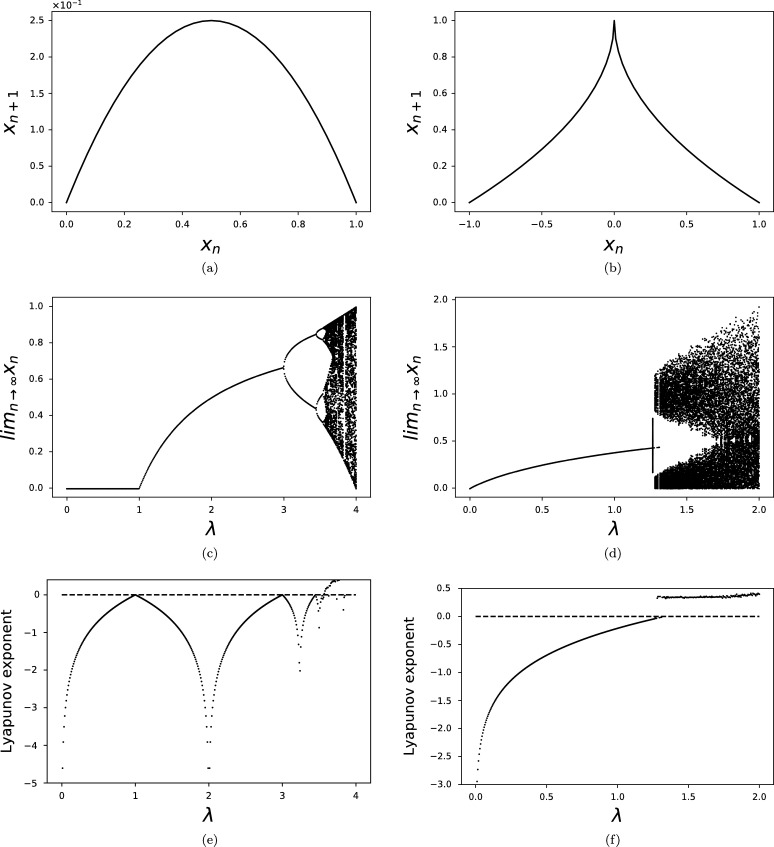
The iteration function, bifurcation diagram and Lyapunov exponent for the logistic map (1) (left column) and the ren map (2) (right column).

To motivate the introduction of a new chaotic map for modelling territorial divisions, we first discuss the logistic map (May, 1976) and its limitations. In Fig. 1(a) we can see the graph of the iteration function for the logistic map:

(1) $$x_{n+1}=\lambda x_{n}(1-x_{n})$$

The bifurcation diagram of the logistic map (1) in Fig. 1(c) illustrates how discrete maps can be useful in modelling the fragmentation of a state. The unique branch that exists for λ<3 can be associated to one undivided territory, while the subsequent branches can represent the different factions that emerge. In our case, the values $x_{n}$ of the map correspond to the area of the territory, but other quantitative measures can be used depending on the available data, such as population, number of cities, army size or any other additive metric for the characteristics of a polity.

Ideally, at any given point in time, the number of branches should correspond to the number of existing polities and the value of each branch should correspond to the area of a territory. While such an exact fit would be desirable, it is difficult to find a model with all the necessary properties to achieve this. For the logistic map, but also for smooth uni-modal maps, there are two main features that prove problematic, each relating to a Feigenbaum constant (Feigenbaum, 1978).

The first property is the fact that the ratio between the width of a branch and the width of one of its two sub-branches tends a constant α≃2.5029 (Feigenbaum, 1978). This means only certain territorial areas can emerge from the bifurcation that are related according to this scaling relationship. This limits the model's versatility and prohibits the application of the model to diverse sets of data.

To explain to second problematic feature, assume *λ* is drawn at random according to a uniform distribution from the interval [1;4]. Then we would expect that the range of values of *λ* for which two branches appear to be proportional to the time in the historical record for which two major polities existed. And a similar property should hold for any number of branches. But, each succeeding period doubling occurs after intervals of decreasing length, with the intervals between each bifurcation decreasing in length by a factor that tends towards the constant δ≃4.6692 (Feigenbaum, 1978). Hence, periods with several co-existing states (e.g., four) should exist for a relatively small fraction of time, which is inconsistent with the historical record.

To overcome some of these limitations, we propose a model based on a map that does not fall in the same universality class as the logistic map. The map we consider is the following:

(2) $$x_{n+1}=\lambda |1-\sqrt{\left|x_{n}\right|}|$$

The graph of the iteration function for is shown in the Fig. 1(b) and resembles the Chinese character ren, which means “person”.^1^ The functions $\lambda |1-|x{|}^{\mu }|$ where μ<1 have similar graphs and to the best of our knowledge they are not known in the mathematics literature under any specific name. In this paper we refer to the maps with these iteration functions as ren maps, and we use this name when referring to example (2).

Fig. 1(d) shows the bifurcation diagram for the ren map (2), and two main types of attractors can be seen: a stable fixed point is reached for $\lambda <\lambda_{c}=4/3$, and chaos for larger values of *λ*, as the Fig. 1(f) of the Lyapunov exponent confirms. The ren map is thus in a different universality class than the logistic map and the scaling properties pertaining to the Feigenbaum constants for smooth maps to not apply. The dichotomous nature of the bifurcation diagram of the ren map makes it well suited for modelling the territorial dynamics of the Chinese dynasties, which have oscillated between periods with a single dominant dynasty, and periods with multiple competing factions, see Table 1.

**Table 1:** The different periods of Chinese history ordered chronologically and classified according to their territorial integrity (Meskill, 1965; Taagepera, 1979, Taagepera, 1997; Song, 1994; Gernet, 1996; Dardess, 2010; Von Glahn, 2016). The average duration of a historical period in the imperial era is approximately 350 ± 50 years.

Period	Time range	Duration (years)	Type
Western Zhou	1045 BCE - 771 BCE	274	Unified
Spring and Autumn Period	771 BCE - 476 CE	295	Intermediate
Warring States and the Qin Dynasty	476 BCE - 206 CE	270	Divided
Han Dynasties	202 BCE - 220 CE	422	Unified
Period of Disunion and Sui Dynasty	220 CE - 618 CE	398	Divided
Tang Dynasty	618 CE - 907 CE	289	Unified
Five Dynasties and Ten Kingdoms, Tripartition	907 CE -1271 CE	364	Divided
Yuan Dynasty and Ming Dynasty	1271 CE - 1636 CE	365	Unified
Qing Dynasty	1636 CE – 1912 CE	276	Unified

While our proposed map (2) addresses some limitation of the logistic map (1), there remains one problematic aspect: half of the values in the chaotic orbits are above the maximum value on the fixed-point branch. The logistic map was originally applied to study population oscillations (May, 1976), in which case the interpretation allows for larger values after a bifurcation. But, in our interpretation this means states can separate into components larger than the original entity, which appears inconsistent. Nevertheless, the separated polities could invade or unite with other territories and exceed the size of the original state. While in reality there are geographical and political limitations to this, the model does not a prior exclude such possibilities. For example, the upper bounds of the chaotic orbits in Fig. 1(d) allows for the possibility of significant fractions of Asia and beyond to be conquered, which historically has been the case with the Mongol Empire (Taagepera, 1997).

The fixed points for the ren map (2) are given by:

(3) $$x=\lambda \left(1+\frac{\lambda -\sqrt{\lambda (\lambda +4)}}{2}\right)$$

The fixed point branch ends at $\lambda_{c}$, which can be determined using (3) and solving the equation $|f^{\prime }(x)|=1\to 3\lambda^{2}+8\lambda -16=0$, where *f* is the iteration function. If a single dominant, unified territory exists at a given time of area *x*, then the value of *λ* can be easily inferred:

(4) $$\lambda =\frac{x}{1-\sqrt{x}}$$

where *x* has been scaled such that it can match a value on the single, fixed-point branch in Fig. 1(d).

## 4 Results

In the case of biological models, the value $x_{n}$ of a map represents the uniquely existing value at index *n* that represents the number of discrete time steps. This means that the attractor exists and at each time instance we measure one value approximating one point on the attractor. To apply chaotic maps to territorial division a different interpretation is necessary due to multiple co-existing polities over any given time period. In fitting equation (2) to historical data, there is a degree of freedom that needs to be set: the scaling of the index *n* with time *t*. Namely, the discrete index *n* can represent, decades, years, months or even days. As long as the timescale of the discrete time step of the index *n* is sufficient small compared to the finest temporal resolution in the data set, this is enough to mimic simultaneity in the real data.

Hence, provided the time step is small enough compared to the timescales in the data, then we can effectively consider multiple points on the chaotic attractor to occur in the same time-frame. Hence, because the time resolution of the data is not fine enough to discern yearly information, but at most at a timescale of several years, then as an idealization we can assume that multiple points on the attractor are accessible over the short term. We assume that both the scaled territorial area *x* and *λ* depend on time:

(5) $$x_{t+1}=\lambda_{t}|1-\sqrt{\left|x_{t}\right|}|$$

where *t* takes on discrete year values. Then, at any given time a unique $x_{t}$ value exists but is not directly accessible via measurement due to low time resolution of the data. Nevertheless, over longer time intervals of several years a spread of value is observable corresponding to the different polities.

Thus, in interpreting the fit of model (5) to the archaeological record we should consider its values bracketed in time intervals of several years. This allows us to meaningfully interpret multiple existing points on the attractor in the same time interval. We note that the (scaled) area does not adjust instantaneously to the steady state. As long as the discrete time step *n* of the map $x_{n}$ corresponds to a small enough real time step (e.g., one year), then within the timescales of the data (typically decades) the map's orbit can approximate the attractor.

To recover the trends in the historical record that are highlighted in Table 1, we determine the evolution of the *λ* parameter over time. For simplicity, we consider an approximation over continuous time λ(t) and recover the discrete $\lambda_{t}$ version by restricting to yearly values. Given the ren map (2) and its bifurcation diagram in Fig. 1(d), we make the natural assumption that the periods of unity in Table 1 correspond the lower values of *λ* (left of the bifurcation), while periods of division and instability correspond to higher values of *λ* (right of the bifurcation). Hence, we expect that the more unified a territory is the smaller the value of *λ* is (the further away it is from the bifurcation). Due to this reasoning, we consider the minima of *λ* to correspond to the maximum area of the stable dynasties. Using equation (4), the parameter *λ* can be determined uniquely for each historical period with a stable, dominant dynasty.

**Figure 2:**
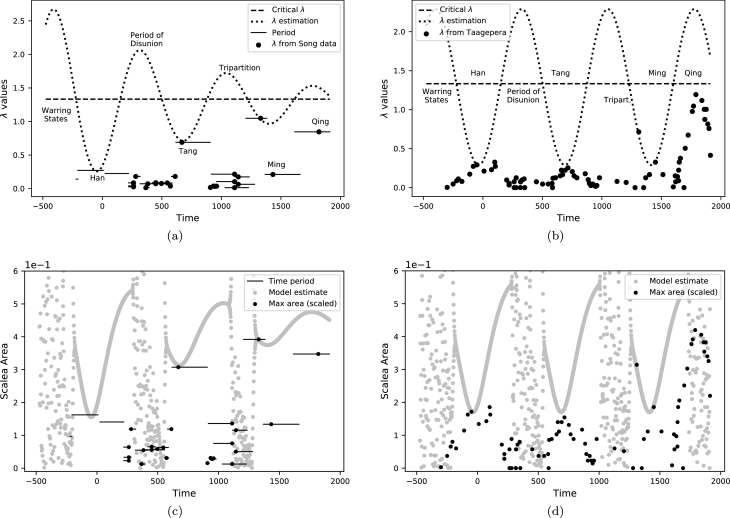
(Top) The evolution of *λ* (dotted) that we determined according to the data sources (Song, 1994; Taagepera, 1979, Taagepera, 1997). The *λ* for the dynasties (dots) indicates a single dominant dynasty in the stable periods (Han, Tang, Ming, Qing) and several in the chaotic periods. The Warring states period, the Period of Disunion, the period after the fall of the Tang and the Tripartition, all correspond to values of *λ* in the chaotic regime, above *λ*_*c*_ = 4/3 (dashed line). (Bottom) The corresponding evolution (grey dots) of the ren map in model (5) for *λ* values determined above. We compare the prediction from (5) with the maximum area for the dynasties (black dots) throughout their lifespan (solid line). The evolution of *λ* and the corresponding map values according to model (5) show a departure from the archaeological record starting with the Ming or Qing dynasties.

Figs. 2(a), (b) show the evolution of the parameter *λ* given the historical record (Song, 1994; Taagepera, 1979, Taagepera, 1997) of territorial changes of the Chinese dynasties during the imperial period 221 BCE-1912 CE. In Figs. 2(c), (d) the areas have been divided by a common factor of $A=3500\times 10^{4}$ km^2^ to fall within the range of values of the fixed-point branch in Fig. 1(d) and the values for the corresponding *λ* are determined using equation (4). The factor *A* is determined using equation (4) by mapping the largest territorial expanse in the data set to be 90% of $\lambda_{c}$. The historical information does not provide a fine-grained evolution of territorial evolution but only captures significant changes over time. Nevertheless, it is sufficient to obtain an overall trend of the major transitions from periods of unity to disunity.

The oscillatory behaviour in *λ* is to be expected due to the inherent seasonality of the data, see Table 1. Finding an equation for the evolution of *λ* requires addressing three aspects: (a) the periodicity, (b) the phase (or time) shift and (c) the evolution of the amplitude. Regarding aspects (a) and (b), the historical data (Taagepera, 1979, Taagepera, 1997; Song, 1994) is largely univocal. As Table 1 shows, the characteristic timescale of a stable or chaotic regime is between 300 to 400 years with an average of 350 years and a standard deviation of 50 years. This implies a period *T* of 700 to 800 years, and the best fit we achieve is for T=725 years (the time span between the start of a stable regime and the next stable one).

The time shift is chosen such that the minima of *λ* occur when the stable dynasties achieve maximum area, dates on which the data sources (Taagepera, 1979, Taagepera, 1997; Song, 1994) are consistent. The only point of significant divergence in the data is regarding (c), the amplitude of oscillations, which depends on the territorial extent of the different dynasties. The general form we posit for the parameter *λ* over time is:

(6) $$\lambda (t)=c+e^{-\gamma t}\sin[\omega (t-t_{0})]$$

where the sinus function captures an oscillatory behaviour, the exponential decay can account for changes in amplitude and *c* is a constant. We see that *λ* oscillates over time below and above the critical value, depending on the territorial integrity of the dynasties in each historical period, and depending on the data source the amplitude of the oscillations either decreases over time, as in Fig. 2(a), or stays constant, see Fig. 2(b). For λ(t) in Fig. 2(a) we determined the following parameter values: $c=1.29,\frac{1}{\gamma }=1250$ years, $\omega =\frac{2\pi }{T}$ where T=725 years and $t_{0}=145$ years. For Fig. 2(b) the parameters c,ω and $t_{0}$ are the same, but γ=0.

## 5 Discussion

As Fig. 2 shows, the ren map (2) gives a coarse model for the territorial dynamics with two main types of regimes: stable dynasties, modelled as stable fixed points and unstable periods, modelled as chaotic attractors. The pattern in Fig. 2(a) from one data source (Song, 1994) reflects a general tendency over time towards imperial unity (Dardess, 2010), while the in Fig. 2(b) the data (Taagepera, 1979, Taagepera, 1997) indicates a unchanging cycle. Nevertheless, there are commonalities and equation (6) is general enough to capture both patterns.

The behaviour of *λ* in Figs. 2 (a), (b) can be interpreted in light of the bifurcation diagram in Fig. 1(d), with *λ* oscillating above and below the critical value $\lambda_{c}=4/3$. How can we interpret the values of the map $x_{n}$ in (5)? If we consider $s_{n}$ to be (scaled) area of the dominant dynasties in Figs. 2 (c), (d), we can then see that $x_{n}-s_{n}$ gives an estimate of the total area of the competing polities. When $x_{n}-s_{n}=0$, then there are no competing polities and $x_{n}$ gives the largest area of the dominant dynasty. Towards the end of a major dynasty, there are several competing forces and the main dynasty is losing territory, with $x_{n}-s_{n}$ increasing and reaching a maximum just before a period of division starts, as can be seen in Figs. 2(c), (d). While the fit for the early stable dynasties is reasonable, the chaotic regimes and later dynasties require further discussion.

During periods of unrest, the appearance of polities and their dissolution was a continuous process and their boundaries were often changing (Lattimore, 1951; Gernet, 1996). Furthermore, the delimitation of the different territories is not exact, and sources differ in their estimation (Song, 1994; Taagepera, 1979, Taagepera, 1997). Based on historical contingencies, in any given period, a different number of factions of different sizes could have emerged and the historical record might have looked vastly different. As such, focusing on a precise number of polities and determining an exact area for each of them is not necessarily consistent with the volatile nature of politics and warfare over time.

Given that territorial boundaries were often shifting and states appearing and disappearing, modelling the unstable periods as chaotic is reasonable. Polities of any given size could have appeared which is reflected in the dense mass of points of the chaotic attractor, see Fig. 1(d) and Fig. 2 (c), (d). In Fig. 2 (c), (d) the minimum values of the ren map correspond to the largest territorial extent of the major dynasties enjoyed at their most stable time. In Fig. 2 (c) the time periods corresponding to the different dynasties have been included, while in Fig. 2 (d) the territorial evolution of the major dynasties is more fine grained and shows an antithetical relationship to the evolution of model (5). This is to be expected as a loss of territory is accompanied by increased instability, eventually leading to a period of division and turmoil.

Should more periods be considered stable? While certain short-lived periods of unity existed, such as during the Qin (221-206 BCE) or Sui (581-618 CE) dynasties, it is questionable if they are best represented by a stable fixed point of the map or a point on the chaotic attractor. Another possible point of contention is how to model the Song dynasty. Due to shifting boundaries and given the constant tensions with competing forces such as the Xi Xia and Liao, and later the Jin, we considered that the period is best modelled as part of a chaotic attractor.

We see from Fig. (2) that *λ* is low during periods of unity and stability and high in unstable, chaotic periods, which implies an antithetical relationship with asabiya, which represents group solidarity. As such, we can define asabiya over time a(t) as follows:

(7) a(t)=C−λ(t)

where *C* is an arbitrary constant. Because λ(t) is at a minimum when dynasties are most stable, equation (7) implies asabiya is at a maximum which accords with historical intuition. Thus, given the properties of λ(t), a(t) behaves as expected, high in periods of unity and decreasing during state fragmentation. Due to equations (6) and (7), we can write down the following equation for asabiya:

(8) $$\overset{¨}{a}+b\overset{˙}{a}+ka-F=0$$

which is the equation of a damped harmonic oscillator. The coefficients are $b=2\gamma ,k=\omega^{2}+\gamma^{2}$ and F=k(C−c), which implies that the oscillator is under-damped in Fig. 2(a) or undamped (b=0) in Fig. 2(b). A better fit to the historical record can be obtained using equation (8) by adding exogenous forcing terms on the right hand side, to capture factors and political decisions that deviate from the prior trend. Thus, deriving equation (8) opens the possibility to justify additional changes and parameters to equation (6).

The equation (8) for asabiya has been derived from the archaeological record and depends on the properties of the ren map (2), specifically on how the transition to chaos occurs. The nature and derivation of the equation contrasts sharply with prior research that posited the Verhulst logistic equation (Bacaër, 2011) for asabiya (Turchin, 2018), which cannot by itself generate oscillations as seen in the historical record.

The fit in Fig. 2 extends backwards in time and at least qualitatively matches what we expect for the Warring States period, namely a high value for corresponding to a chaotic regime. The evolution of λ(t) shows secular cycles (Turchin and Nefedov, 2009) consistent with the historical record for over 1800 years but diverges from it starting with the Ming dynasty in Fig. 2(a), and the Qing dynasty in Fig. 2(b). While these observations open a broad topic for discussion, we only note that the breaking of the patterns can be related to the markedly conservative nature of the Ming policies, and the long reign of Emperor Kangxi (1661-1722) that contributed to stabilising the Qing period.

**Figure 3:**
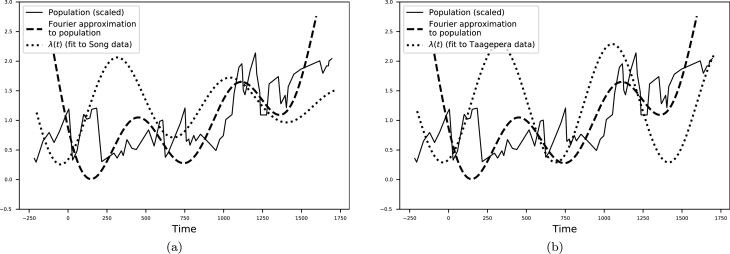
The population data (Zhao and Xie, 1988) (solid line) is divided by *P* = 50 million people and the 3rd order Fourier series approximation (dashed line) is computed. We compare the evolution of *λ* (dotted line) with the population trends.

On the other hand, why does the trend in λ(t) persist for 1800 years? Analysis of Chinese population data (Turchin, 2018) also reveals a characteristic timescale for oscillations of 300 to 400 years, similar to our estimate from Table 1. In Fig. 3 we compare the evolution of *λ* with the population and a low order Fourier approximation, with frequencies comparable to *ω* in equation (6). We employed the Fourier approximation to determine long-term patterns in the data and to filter out noise. We see that population maxima / minima are reached in the periods when λ(t) has a markedly negative / positive slope. This implies asabiya, defined in equation (7), is increasing / decreasing at population maxima / minima, which is consistent with historical intuition. Furthermore, it suggests there is a second-order feedback process (Turchin, 2018) relating the population and changes in asabiya, similar to how the concavity of a function relates to its second derivative.

Thus, the oscillatory, persistent pattern in *λ* can be attributed to multiple feedback mechanisms operating over the long-term and that involve population dynamics, state apparatus, territorial changes and other factors posited by structural demographic theory (Turchin and Nefedov, 2009; Goldstone, 2016). In addition, sunk-costs effects (Janssen et al., 2003) can contribute to long-term societal developments and transition from stability to division or collapse. While an extended model incorporating multiple feedback loops is desirable, this is beyond the scope of the present paper and is the aim of future work.

## 6 Conclusions

In the present work, we have aimed at providing a framework for modelling state fragmentation and unification using certain mathematical methods, namely discrete maps. As such, we discussed one of the most well-known maps, namely the logistic map and how bifurcations can model state divisions, along with some limitations that all smooth maps share. Due to these constraints, we proposed a model from a new class of maps, which we refer to as ren maps, that can provide a more robust, coarse description of territorial dynamics.

The focus of our modelling has been on reproducing the archaeological records (Taagepera, 1979, Taagepera, 1997; Song, 1994; Tan, 1982) regarding territorial changes for the Chinese dynasties. We reproduced the archaeological record regarding the territorial extent of the dynasties using the ren map, which, depending on a parameter *λ*, shows transitions to order / chaos in line with historical data. Given the antithetical nature of the parameter to the degree of territorial unity, we then defined asabiya as a constant minus *λ* (the constant is an arbitrary reference level). From this definition, we determined an equation of asabiya, which is that of a harmonic oscillator, and can be damped or undamped. This finding contrasts with prior work that modelled asabiya starting from the Verhulst logistic equation (Turchin, 2018).

The historical pattern of asabiya shows secular cycles (Turchin and Nefedov, 2009) consistent with 1800 years of Chinese history, from the Warring State periods to the beginning of the Ming or Qing dynasties, depending on the date sources used (Song, 1994; Taagepera, 1979, Taagepera, 1997). Furthermore, by comparing the evolution of *λ* with population data (Zhao and Xie, 1988), we see a pattern emerging where the largest changes in asabiya occur at extreme values of the population. Overall, we contribute to the literature that uses dynamical systems to model societal developments by extending existing methods that rely on ODEs (Roman et al., 2017, Roman et al., 2018; Roman and Palmer, 2019) to discrete maps.

## Declarations

### Author contribution statement

Sabin Roman: Conceived and designed the experiments; Performed the experiments; Analyzed and interpreted the data; Contributed reagents, materials, analysis tools or data; Wrote the paper.

### Declaration of interests statement

The authors declare no conflict of interest.

### Data availability statement

Data associated with this study has been deposited at GitHub under the accession number https://github.com/sabinroman0577/historical-dynamics-chinese-dynasties.

### Funding statement

This work was supported by 10.13039/100008118Grantham Foundation for the Protection of the Environment.

### Additional information

No additional information is available for this paper.

## Footnotes

1 In addition, Confucian philosophy associates the notion of ren with empathy and “how two people should treat one another”.
